# Genome-wide gene expression analysis supports a developmental model of low temperature tolerance gene regulation in wheat (*Triticum aestivum L*.)

**DOI:** 10.1186/1471-2164-12-299

**Published:** 2011-06-07

**Authors:** Debbie Laudencia-Chingcuanco, Seedhabadee Ganeshan, Frank You, Brian Fowler, Ravindra Chibbar, Olin Anderson

**Affiliations:** 1 Genomics and Gene Discovery Unit, USDA-ARS WRRC, 800 Buchanan St. Albany CA 94710 USA; 2 Department of Plant Sciences, University of Saskatchewan, 51 Campus Drive, Saskatoon, Saskatchewan S7N 5A8 Canada; 3 Department of Plant Science, University of California, One Shields Avenue, Davis, CA 95616 USA

**Keywords:** wheat, transcriptional profiling, low temperature adaptation, cold acclimation, vernalization, microarrays

## Abstract

**Background:**

To identify the genes involved in the development of low temperature (LT) tolerance in hexaploid wheat, we examined the global changes in expression in response to cold of the 55,052 potentially unique genes represented in the Affymetrix Wheat Genome microarray. We compared the expression of genes in winter-habit (winter Norstar and winter Manitou) and spring-habit (spring Manitou and spring Norstar)) cultivars, wherein the locus for the vernalization gene *Vrn-A1 *was swapped between the parental winter Norstar and spring Manitou in the derived near-isogenic lines winter Manitou and spring Norstar. Global expression of genes in the crowns of 3-leaf stage plants cold-acclimated at 6°C for 0, 2, 14, 21, 38, 42, 56 and 70 days was examined.

**Results:**

Analysis of variance of gene expression separated the samples by genetic background and by the developmental stage before or after vernalization saturation was reached. Using gene-specific ANOVA we identified 12,901 genes (at *p *< 0.001) that change in expression with respect to both genotype and the duration of cold-treatment. We examined in more detail a subset of these genes (2,771) where expression was highly influenced by the interaction between these two main factors. Functional assignments using GO annotations showed that genes involved in transport, oxidation-reduction, and stress response were highly represented. Clustering based on the pattern of transcript accumulation identified genes that were up or down-regulated by cold-treatment. Our data indicate that the cold-sensitive lines can up-regulate known cold-responsive genes comparable to that of cold-hardy lines. The levels of expression of these genes were highly influenced by the initial rate and the duration of the gene's response to cold. We show that the *Vrn-A1 *locus controls the duration of gene expression but not its initial rate of response to cold treatment. Furthermore, we provide evidence that *Ta.Vrn-A1 *and *Ta.Vrt1 *originally hypothesized to encode for the same gene showed different patterns of expression and therefore are distinct.

**Conclusion:**

This study provides novel insight into the underlying mechanisms that regulate the expression of cold-responsive genes in wheat. The results support the developmental model of LT tolerance gene regulation and demonstrate the complex genotype by environment interactions that determine LT adaptation in winter annual cereals.

## Background

Low temperature (LT) is a major abiotic stress that limits the growth, productivity and geographical distribution of agricultural crops. Even in established agricultural production areas, seasonal or episodic freezing events can lead to significant crop loss. In the U.S., ten percent of the sources for claims filed under the Federal crop insurance since 1988 have been due to frost, freeze and cold weather http://www.ers.usda.gov. Clearly, the development of crops with better adaptation to cold could save billions of dollars. Crops with improved LT tolerance also have a role to play in the goal towards multiple land use and sustainable production systems. Greater LT tolerance allows more flexibility in farm management choices thereby increasing the opportunity to create more environmentally friendly production systems, reduce herbicide costs, increase crop moisture utilization, lower energy requirements, and increase productivity.

Identifying and understanding the mechanisms of LT adaptation is crucial to the development of cold-tolerant crops. The Triticeae tribe of the grass family includes some of the most cold-hardy winter annual plants. Wheat, barley, and rye are grown in temperate regions throughout the world and account for a third of the total world grain. The ability to survive freezing within each of these species differs significantly - some cultivars can survive winter (cold-hardy winter cultivars) in temperate climates while others cannot (cold-sensitive spring cultivars) - making members of the Triticeae tribe an excellent system in which to study LT adaptation in plants.

The two major loci that control LT tolerance in Triticeae are the frost resistance *Fr-1 *and *Fr-2 *[1]. The *Fr-1 *locus is tightly linked to the vernalization locus *Vrn1*, the major regulator of the transition of the vegetative to reproductive meristem in response to cold treatment. Allelic variations in the *Vrn1 *locus are the main determinants of the winter and spring habit in wheat and barley [2,3]. The *Vrn1 and Fr-1 *loci have not been genetically uncoupled, and are hypothesized to be the same locus [4]. The pleiotropic effects of the *Vrn1 *locus can explain most of the associated LT tolerance and winter habit [4,5]. Thus, for this paper we will refer to this locus as *Vrn1*. The *Fr-2 *locus contains a cluster of C-repeat binding transcription factors (CBFs), known to be involved in the regulation of cold induced genes in several plant species including members of the Triticeae tribe [6,7]. Since both the *Vrn1 *and *Fr-2 *loci are located in the long-arm of chromosome 5 in wheat, the interpretation of data from previous experiments comparing the response of spring and winter genotypes to cold treatment do not separate the effect of variations at the *Vrn1 *and *Fr-2 *loci [8,9].

Phenotypic studies in Triticeae have shown that LT-induced protective mechanisms are developmentally regulated and involve acclimation processes that can be stopped, reversed and restarted [10]. Several studies have shown that the transition of the shoot apex from vegetative to reproductive meristem is highly correlated with a decline in the ability to induce LT tolerance upon cold treatment [11,12]. This observation was incorporated in a developmental model of LT tolerance gene regulation [10] wherein the genes that determine the length of the apical meristem vegetative stage act as switches that control the duration of expression of LT tolerance genes [13,14] and a rate component, which is related to the differences in threshold induction temperatures [15], determine the degree that these genes are up regulated. The model contends that the full expression of cold hardiness genes only occurs in the vegetative stage. Thus, plants that are already in the reproductive phase have a limited ability to re-acclimate following periods of exposure to warm temperatures. The model predicts that genotypes with longer vegetative stage are more responsive to extended periods of temperatures in the acclimation range, hence, have higher potential to develop LT tolerance.

The availability of a set of reciprocal near-isogenic lines (NILs) for the *Vrn-A1 *locus of a tender spring habit (cv Manitou - *Vrn-A1*) and a cold hardy winter habit (cv Norstar - *vrn-A1*) genotype [16,17] provided an opportunity to design experiments that determine the effect of the *Vrn1 *locus on the rate and the duration components of the developmental model for LT tolerance gene regulation without the confounding effect of the *Fr-2 *locus. In this paper, the phenotypic response of these lines to cold treatment was investigated utilizing microarray analyses to identify and assess the relative expression patterns of cold-responsive genes and their association with the development of LT tolerance. We report on the impact of the *Vrn-A1 *locus on global gene expression during vernalization and LT acclimation and compared LT induced gene expression in tender and cold hardy genetic backgrounds when the effect of *Vrn-A1 or Fr-2 *locus has been neutralized. We focused on a set of genes whose expression shows strong interaction between genotype and duration of cold treatment and assessed their potential role in vernalization and cold acclimation. Our data provided a strong molecular support for the developmental model of LT tolerance gene regulation in wheat.

## Materials and methods

### Plant materials

Wheat (*Triticum aestivum *L.) cultivars and near isogenic lines (NILs) used in this study were developed and characterized as previously described [4]. Briefly, the winter cultivar 'Norstar' (*vrn-A1*) and the spring cultivar 'Manitou' (*Vrn-A1*) were crossed to produce an initial hybrid that was then backcrossed 10× to each parent. In subsequent generations, each BCF_1 _of the previous generation was selected for heterozygosity (*Vrn1/vrn1*) at the *Vrn-A1 *locus. When winter Norstar was the recurrent parent, heterozygosity at the *Vrn-A1 *locus was based on the spring habit, which would be *Vrn1/vrn1*, due to the dominance of the spring habit allele, whereas all other progeny would be winter habit. When spring Manitou was the recurrent parent, heterozygosity (*Vrn1/vrn1*) at the *Vrn-A1 *locus was based on the heterozygotes' flowering time, which was several weeks later than that of the homozygous (*Vrn1/Vrn1*) spring habit. This phenotype-based selection ensured that the donor parent allele was incorporated into the genetic background of the recurrent parent.

### Phenotypic studies

For these studies, imbibed seeds were held in the dark for 2 days at 4°C and then transferred to an incubator and held for 3 days at 22°C. Actively germinating seeds were transferred, embryo down, to plexiglass trays with holes backed by a 1.6 mm mesh screen and grown for 10 days in hydroponic tanks filled with continuously aerated one-half strength modified Hoagland's solution[15] at 20°C in 16-hour days at 320 μmol m^-2^s^-1 ^PPFD, by which time they had 3-4 fully developed leaves and visible crowns. These seedlings were then transferred to 6°C chambers (measured at crown level) under 16-hour photoperiod and 220 μmol m^-2^s^-1 ^PPFD and sampled at regular intervals. The experimental design was a 4 (genotypes) × 16 (cold treatment periods: 0, 2, 7, 14, 21, 28, 35, 42, 49, 56, 63, 77, 84, 91 and 98 days) factorial in a randomized complete block design with three replicates.

#### Freeze test response

The LT_50 _(temperature at which 50% of the plants are killed by LT stress) of each genotype at the end of each LT acclimation period was determined using the procedure outlined by Fowler, 2008 [15]. Crowns were covered in moist sand in aluminum weighing cans and placed in a programmable freezer that was held at -3°C for 12 h. After 12 h they were cooled at a rate of 2°C h^-1 ^down to -17°C, then cooled at a rate of 8°C h^-1^. Five crowns were removed from the freezer at 2°C intervals for each of five test temperatures selected for each genotype in each treatment. Samples were then thawed over night at 3°C. Thawed crowns were transplanted into 52 × 26 × 6 cm black plastic trays (Kord Products, Bramalea, ON, Canada) containing "Redi-earth" (W. R. Grace and Co. of Canada Ltd., Ajax, ON, Canada) for re-growth. The trays were placed in a growth room maintained at 20°C with a 16-hour day and 4-hour night. Plant recovery was rated (alive vs. dead) after 3 weeks and LT_50 _was calculated for each sample.

#### Flowering time response

Time to flowering was estimated using the final leaf number (FLN) procedure previously described [16]. The time when the final leaf is produced indicates the period that the shoot apical meristem has transitioned from vegetative to a reproductive phase. Germinated seeds exposed to 6°C as outlined above were planted in pots (2 plants/pot) and transferred weekly to 20°C chambers under conditions favoring floral induction (20°C, 16-hour photoperiod). The tops of the pots were wrapped in aluminum foil to minimize radiant heat absorption from the lights and the plants were uniformly fertilized with "Osmocote" (Chisso-Asahi Fertilizer Co., Tokyo, Japan) sustained-release fertilizer and a nutrient-complete ("Tune-up" TM, Plant Products Ltd., Brampton, ON, Canada) water-soluble solution as required. Leaves were numbered and the plants were grown until the flag leaf emerged and the FLN on the main shoot could be determined. Saturation of the vernalization requirement was considered complete for each genotype once the cold treatment no longer reduced its FLN.

#### Shoot apical meristem (SAM) development

The stage of SAM development during cold treatment was determined by dissection of a minimum of three shoot apices from the main stem of plants grown at 20°C (floral inductive condition) for 10 or more days after they were removed from vernalization at 6°C. The mean number of days to double ridge formation (marker of a reproductive meristem) was recorded for the dissected apices per time point.

### Global gene expression profiling

The experimental design for the microarray studies included 4 genotypes (winter Norstar and spring Norstar NIL and spring Manitou and winter Manitou NIL) acclimated at 0, 2, 14, 21, 35, 42, 56, and 70 days. Crown tissue was harvested after each acclimation period at the same time each day to neutralize circadian rhythm effect. RNA was isolated from three biological replicate samples (pool of 25 crowns/sample) for each acclimation period.

#### RNA extraction

Total RNA was extracted from crown tissues using a modified Trizol method (Invitrogen, Inc., Burlington, Ontario, Canada). Briefly, about 0.5 g of tissue was ground in liquid nitrogen and the powder was mixed thoroughly with 5 mL of Trizol reagent in an RNAse-free petri dish. The slurry was transferred to a 15-mL RNAse-free tube and mixed with an equal volume of chloroform. The mixture was centrifuged at 2700 × g at 4°C for 10 minutes, the supernatant transferred to a new tube and the chloroform extraction repeated once more. Total RNA was precipitated using isopropanol and the pellet briefly dried and re-suspended in 600 μL of RNAse-free water. The total RNA was then quantified on a spectrophotometer and cleaned using the Purelink Micro-to-Midi RNA clean-up kit (Invitrogen, Inc., Burlington, Ontario, Canada) according to manufacturer's instructions.

#### Microarray hybridization and data analysis

The Affymetrix Wheat Genome GeneChip array was used to interrogate the RNA samples. RNA labelling and microarray hybridization were performed according to the manufacturer's instructions (Affymetrix Inc, Santa Clara, CA USA). Microarray data were extracted from scanned GeneChip images and analyzed using the Affymetrix Microarray Suite (MAS) version 5.0. Probe set signal normalization and summarization was carried out using the Robust Multi-array normalization algorithm (RMA), as implemented in GeneSpring software. RMA normalized values were filtered for those present (as determined by the Affymetrix MAS probe summarization protocol) in 2 out of 3 of the biological replicates for each time-point.

The RMA normalized values were used as input in a gene-specific ANOVA and a false discovery rate correction with a cut-off at P = 0.01, was used to identify genes that show differential accumulation during cold treatment. ANOVA was implemented using SAS version 9.0 (SAS Institute). K-means clustering of gene expression profiles was implemented using Genesis [18]. Gene expression values (log 2) were median-centered before clustering using k-means algorithm with 50 iterations, Euclidian distance and 8 clusters. Gene Ontology annotations were clustered using Blast2GO [19]. A list of probesets matching rice and Brachypodium gene loci was generated using HarvEST http://www.harvest-web.org.

For the purpose of this work, a probe set was deemed to represent a potentially unique wheat gene and the accumulation of its transcript as measured by the signal intensities in each probe set represent the "expression" of the gene. The dataset was further filtered to remove cross-hybridizing probes based on the Affymetrix probe set name designation but retaining those that recognizes splice variants of the same gene. The wheat oligoarray database at http://www.plexdb.org was used to verify the most recent annotations for the probesets.

Microarray data have been deposited to the NCBI Gene Expression Omnibus (GEO) database with Accession Number GSE23889.

## Results and Discussion

### 1 Genetic stocks and experimental design

To investigate low-temperature tolerance in cereals we compared the response to cold of four near-isogenic lines (NILs) previously generated [16] from two cultivars, one a non-hardy spring wheat (spring Manitou) and the other a very cold-hardy winter wheat (winter Norstar), wherein the major vernalization locus *Vrn-A1 *was swapped (Table 1). Ten backcrosses to the recurrent parents prior to selfing produced homozygous NILs (spring Norstar and winter Manitou) that are theoretically > 99.95% genetically similar to the parental lines. Based on the location of neighboring markers on chromosome 5AL in a mapping population developed from a winter Norstar × winter Manitou [9], the winter Norstar region *vrn-A1 *locus carried by winter Manitou was estimated to be less than 37 cM [4]. The CBF cluster in the *Fr-2 *locus, which is distally located 30 cM away [15,19], was not included in the swapped *Vrn-A1 *region [4], thus, was fixed in the Norstar and Manitou backgrounds.

**Table 1:** Genotype, LT50 and FLN of near isogenic lines used in these studies

Line	Genotype	LT50	FLN
winter Norstar	vrn-A1, vrn-B1, vrn-D1	-23a	13a
spring Manitou	***Vrn-A1***, vrn-B1, vrn-D1	-8.3c	8c
winter Manitou	vrn-A1, vrn-B1, vrn-D1	-13.3b	10b
spring Norstar	***Vrn-A1***, vrn-B1, vrn-D1	-13b	11b

FLN (final leaf number) indicates the onset of the transition of the apical meristem from vegetative to reproductive stage; LT50 indicates the lowest temperature by which 50% of the treated samples survived after vernalization; a to c - within columns, means followed by the same letter are not different as determined by Duncan's new multiple range test (P < 0.05).

These lines allowed us to design experiments to compare the effect of cold temperature on the phenotype and changes in global gene expression in spring and winter hexaploid wheat without the confounding effects of genetic variations in the *Vrn-A1 *and the *Fr-2 *loci. Comparison between lines with the same genetic background e.g. winter Norstar versus spring Norstar or spring Manitou versus winter Manitou will reveal differentially expressed genes highly correlated with the *Vrn-A1 *locus. Conversely, comparison between lines with similar growth habits e.g. winter Norstar versus winter Manitou or spring Manitou versus spring Norstar neutralizes the effect of the *Vrn-A1 *locus on gene expression.

### 2 Phenotypic response of wheat to cold treatment

**Figure 1:**
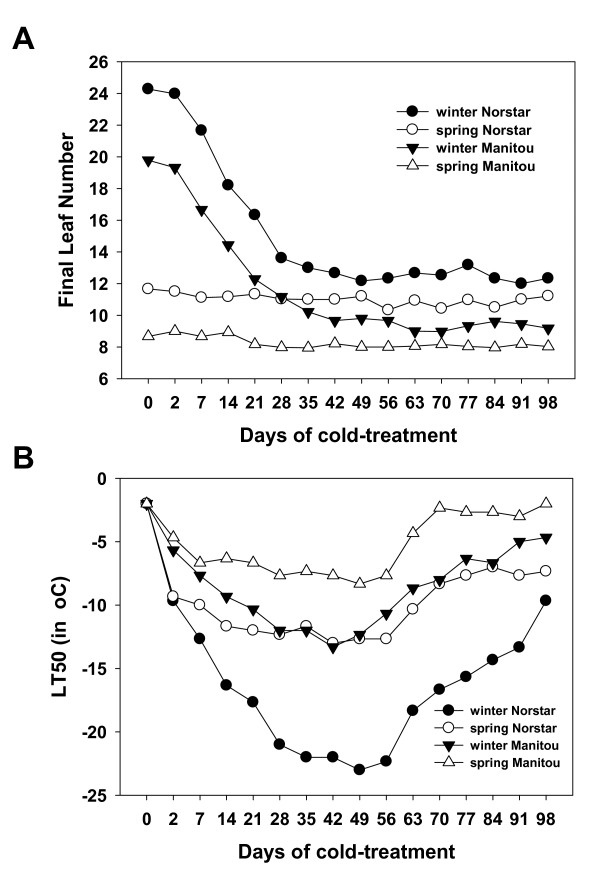
**Phenotypic response to cold-treatment**. A) Final leaf number (FLN) and B) Low-temperature tolerance (LT_50_) of winter Norstar and spring Manitou and the near isogenic lines spring Norstar and winter Manitou acclimated at 6°C for 0 to 98 days (SE of data points = 0.62).

We investigated the phenotypic response to cold of the four genetic stocks we used in this experiment by measuring their final leaf number (FLN) and their tolerance to cold temperature (LT_50_). The parental cultivars, winter Norstar and spring Manitou, and the NILs, spring Norstar and winter Manitou, were evaluated over 16 cold treatment periods at 6°C under a 16-hour (long) day. Analyses of variance showed that genotype, the duration of cold treatment and their interactions were highly significant (P < 0.001) for LT_50 _and FLN indicating that there were measurable differences in the response to cold treatment (Figure 1 and Table 1).

#### 2.1 Vernalization response

Vernalization is the acceleration of the competence to flower by cold-adapted plants upon exposure for a period of time to low non-freezing temperature. In cereals with vernalization requirement, exposure to temperatures in the vernalization range shortens the vegetative phase and decreases the FLN. A vernalization response reduces the risk of winter habit cultivars entering the cold-sensitive reproductive stage until the danger of low temperature damage has passed. Vernalization saturation is the point at which a plant no longer requires further exposure to vernalization temperatures to enter the reproductive phase under warm inductive conditions [16,17].

Vernalization saturation is considered to have been achieved once cold treatment no longer reduces the FLN of a genotype. At this point, the primordia on the shoot apex stop forming leaves and start developing the reproductive structures of the spike when exposed to inductive conditions. Consequently, the time at which the minimum FLN is reached identifies the time when the transition from the vegetative to the reproductive (VRT) phase occurs. Consistent with previous reports [16,17], the changes in FLN in response to cold treatment in the biological material we used showed that the spring habit cultivars do not need vernalization to flower (Figure 1A). The apical meristem of spring Manitou and spring Norstar were already programmed to transition to reproductive stage at 15 days after seed imbibition, even before they were subjected to cold treatment. In contrast, 49 days of cold treatment were required for winter Norstar to reduce its FLN from 24 leaves to 12 while winter Manitou required cold treatment for 42 days to reduce its FNL number from 20 to 9.

**Figure 2:**
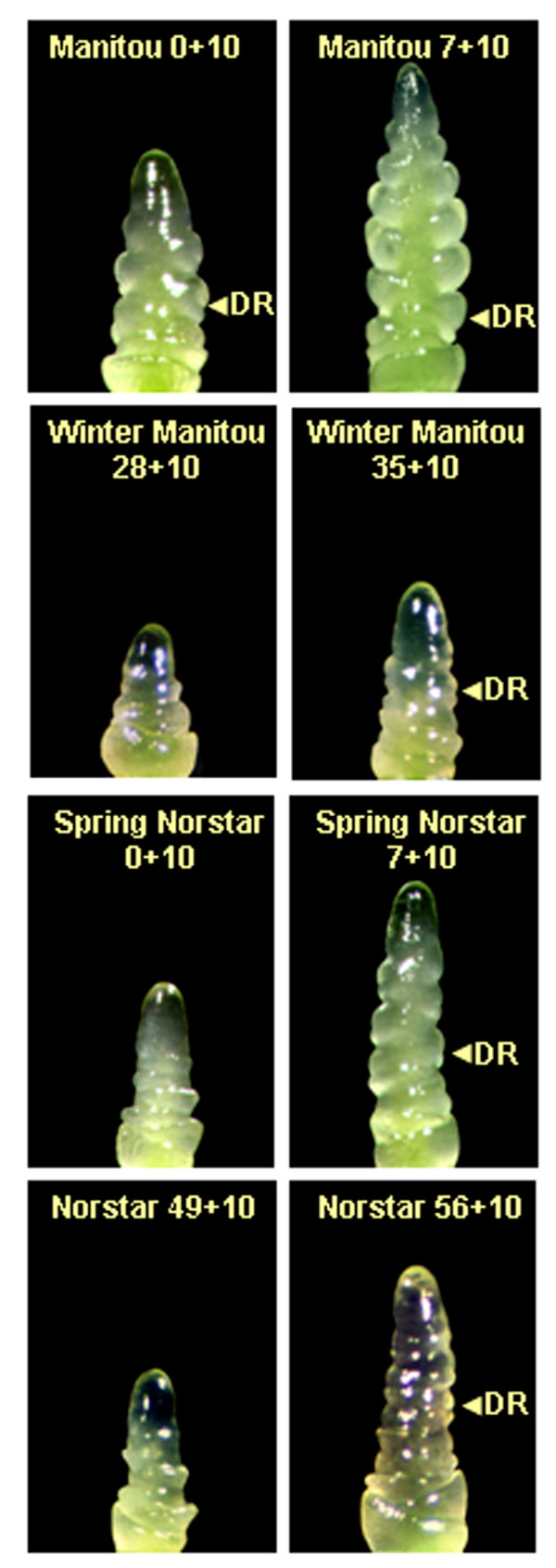
**Shoot apex development in winter Norstar and spring Manitou and the near isogenic lines spring Norstar and winter Manitou**. Day 0 is the start of vernalization/acclimation at 6°C following 2 d growth at 4°C plus 13 d at 20 to 22°C (15 day pre-treatment). First number in each plate indicates the days the plants were grown at 6°C. Second number (10) indicates that the plants were grown an additional 10 days at 20°C before sampling for dissection e.g., 7 + 10 indicates 7 d at 6°C followed by 10 d at 20°C. Arrow indicates double ridge. The apices of samples that bracket the vegetative to reproductive phase transition for each genotype are shown. A) Manitou 0+10 SAM at stage-5; B) Manitou 7+10 SAM at stage-7; C) Winter Manitou 28+10 SAM at stage 0; D) Winter Manitou 35+10 SAM at early stage-5; E) Spring Norstar 0+10 SAM at stage-2 F) Spring Norstar 7+10 SAM at stage-5; G) Winter Norstar 49+10 SAM at stage-2 H) Winter Norstar 56+10 SAM at stage-5. DR, double ridge, was used as a marker of a reproductive meristem.

The changes in the shoot apex morphology in response to cold treatment were also monitored by dissection after seedlings were grown under inductive conditions. As shown in Figure 2, spring Manitou shoot apex developed double ridges, without vernalization treatment, thus, was already competent to flower. However, even though it shares the same vernalization alleles as spring Manitou, double ridge formation was delayed in spring Norstar (Figure 2E), indicating that factors outside of the swapped *Vrn-A1 *locus are involved in the acceleration of competence to flower. In this instance spring Norstar had a minimum FLN that was greater than spring Manitou, which delayed the vegetative/reproductive transition independent of the vernalization gene. In contrast, the meristem of unvernalized winter habit lines remained in the vegetative stage for the duration of the experiment even when grown under inductive conditions (data not shown). It took about 35 days and 49 days of vernalization for the apical meristem of winter Manitou and winter Norstar, respectively, to reach vernalization saturation (Figure 1A).

#### 2.2 Cold-acclimation

Cold acclimation is a process by which increased tolerance to cold is accumulated after a plant is exposed to low non-freezing temperatures. The temperature at which 50% of the plants are killed (LT_50_) by LT stress is used to measure the plants' ability to cold acclimate. The LT_50 _values for the genotypes used in this experiment exhibited a typical curvilinear relationship obtained for these lines [17] with days of acclimation at 6°C (Figure 1B). Spring Norstar became less hardy than winter Norstar, whereas, winter Manitou gained LT tolerance comparable to spring Norstar. The lowest LT_50 _approached -23°C at 49 d for winter Norstar and -13.3°C at 42 d for winter Manitou. These values were achieved at the vegetative to reproductive transition (VRT) stage for these two genotypes as indicated by FLN measurements. Rapid acclimation during the first week was followed by relatively minor increases in the LT tolerance for both spring habit genotypes. Interestingly, while winter Manitou gained 5°C in additional LT tolerance over spring Manitou as a result of the *vrn-A1 *allele, spring Norstar lost 10°C in LT_50 _compared to winter Norstar due to substitution with the *Vrn-A1 *allele. This indicates that gain of the recessive *vrn-A1 *allele had a greater affect on the LT tolerance of winter Manitou than the dominant *Vrn-A1 *allele from spring Manitou had on spring Norstar.

The initial rates of acclimation were more rapid in winter and spring Norstar than those of spring and winter Manitou. The initial *rate *of acclimation was the same for winter and spring Norstar and similar in spring and winter Manitou indicating that factors controlling *rate *of acclimation were located outside the swapped *Vrn1 *locus. All the genotypes begin to de-acclimate after a few weeks of cold-treatment. In the winter genotypes, de-acclimation occurred after VRT.

### 3 Global changes in gene expression in response to cold-treatment

We used acclimated crown tissue of 3-leaf stage plants for the global gene profiling experiment. Although the leaves would have been a more convenient tissue to use, recent experiments have shown that the low-temperature response of the leaves to cold treatment is different from that of the crown at the molecular level [9,20-22]. In overwintering wheat, it is the crown that survives to reestablish the plant roots and leaves in spring following high stress winters when sub-lethal tissue damage has been incurred. Consequently, it is the reprogramming of gene expression to acclimate the crown meristems that is critical to survival under low-temperature stress.

In this study, we compared the global changes in gene expression in the crowns of the cold-hardy and cold-sensitive genotypes in response to cold treatment using the Affymetrix Wheat Genome Chip array. The Affymetrix array contains 61,127 probes representing 55,052 potentially unique genes. The experimental design included three biological samples for each of the 8 cold treatment periods for each of the four genotypes with a total of 96 hybridizations.

**Figure 3:**
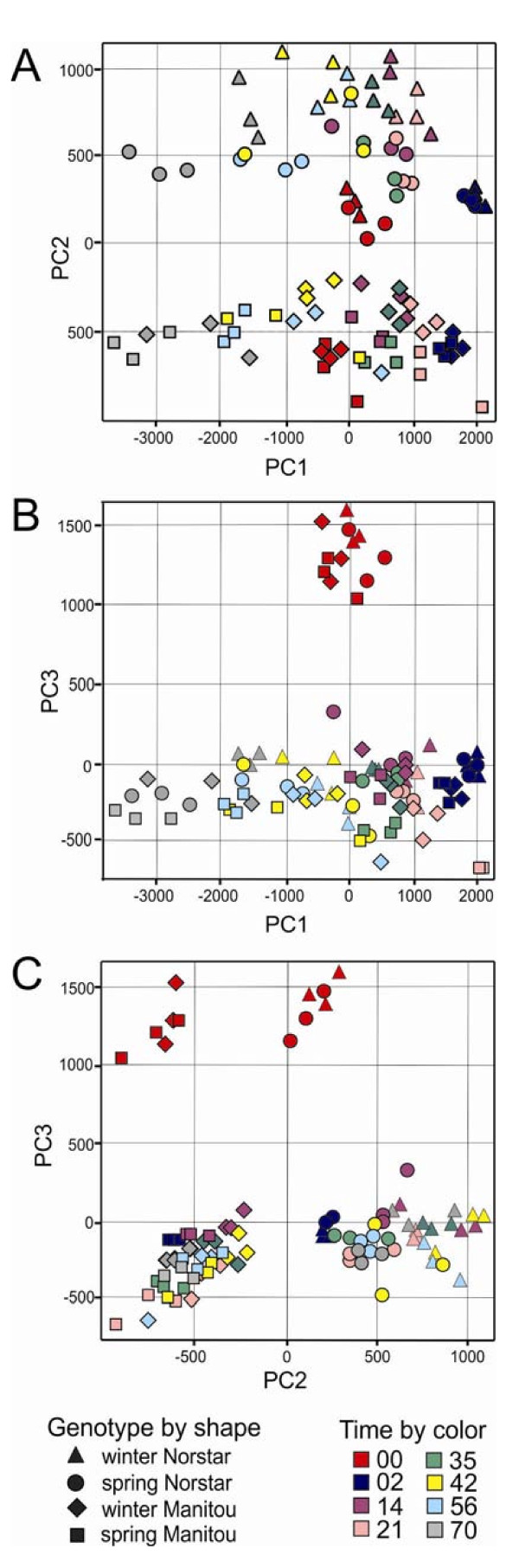
**Principal component analyses**. PC1 (Principal Component 1) separated the samples by the duration of cold-treatment; PC2 separated the samples by genotype and PC3 separated the samples by treatment (cold treated versus untreated). Symbols with the same shape and color represent biological replicates; the numbers on the axes of the graphs refers to the eigenvalues for each component.

Principal component analysis (PCA) was performed to determine the structure of the dataset of almost 6 million data points generated by the experiment. PCA reduces the dimensionality of large data sets and determines the direction of the major variables or components that influence the result of the experiment. PCA analysis indicated that the first three components accounted for 89% of the variability in the data set. As shown in Figure 3, the first principal component (PC1, 49%) separated the data by the duration of cold treatment, PC2 (21%) separated the data by the genotype of the biological samples and PC3 (19%) separated the data by treatment (cold-treated versus untreated). Thus, PCA identified the three main factors that were manipulated in the experimental design. The biological replicates clustered together indicating the reproducibility of the sampling method. For further analysis, the dataset was filtered for genes expressed in at least two of the three biological samples per time-point (= 39,288 genes). After further filtering out of probesets that may cross-hybridize with other genes based on the Affymetrix probe nomenclature, 52% (= 31,768) of the probesets were used for detailed analysis.

**Figure 4:**
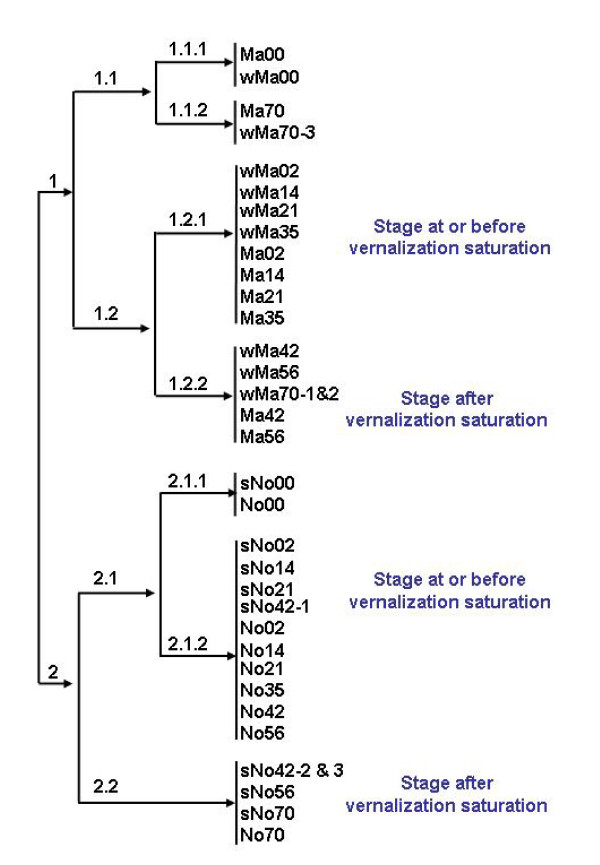
**Correlation of variance of gene expression between samples**. Dendogram for the first 2 levels of branching of a 96 × 96 sample comparison matrix (see Additional file 1).

Correlation of variance between samples was determined to provide insight into the relationships among changes in gene expression. The 96 × 96 sample comparisons resulted in a matrix with a highly branched dendogram (see Additional file 1). The first level of branching separated the samples by genotype (Figure 4) where Cluster 1 contained all the samples for spring and winter Manitou and Cluster 2 contained all the samples for winter and spring Norstar. Cluster 1 was further subdivided into two sub-clusters. Cluster 1.1 grouped the unacclimated treatments (zero days) together with the 70-day acclimated treatments that were mainly from spring Manitou indicating that the gene expression in spring Manitou acclimated for 70 days was more similar to the unacclimated samples. Cluster 1.2 grouped together all the cold treated samples with a spring Manitou background except for wMa70-3. Cluster 1.2 further separated the winter and spring Manitou samples into before or after cold de-acclimation, which coincided with the vegetative/reproductive transition (VRT) point in vernalization requiring winter Manitou. Similarly Cluster 2, which contained all the sample dates for winter and spring Norstar, was subdivided into two with Cluster 2.1 containing all the stages before cold de-acclimation, which coincided with VRT in winter Norstar and Cluster 2.2 those after VRT.

LT studies have previously shown that wheat has only limited capacity to re-acclimate when transferred to acclimating temperatures after the plants have entered the reproductive stage [23]. Our results provide molecular evidence for the difference in gene expression between these two states of the meristem development. More in-depth analysis of the differentially expressed genes before and after VRT could help in the identification of the primary factors involved in the regulation of both vernalization and cold acclimation.

#### 3.1 Identification of differentially expressed genes

We used gene-specific ANOVA [24,25] to identify which of the 31,768 genes were differentially expressed among genotypes during cold acclimation and vernalization. In the ANOVA model, genotype and the duration of cold treatment accounted for 33.51% and 53.41% of the variation in gene expression, respectively. The interaction between genotype and duration of cold-treatment also contributed significantly at 7.43% (p < 0.01) while the remaining sources of variation (residual error) only accounted for 0.05% of the total variation.

As shown in Table 2, 84% of the expressed genes (26,646 at p < 0.01) showed differential expression during the course of 70-day cold treatment. Thus, more than 48% of the genes represented on the array were cold responsive. Even with a high confidence level (*p *< 0.001), 78% of the expressed genes (24,670) varied in expression with respect to the duration of cold treatment and 46% (14,617) varied in expression with respect to genotype. Around 41% of the genes (12,901) significantly varied in expression with respect to *both *the duration of treatment and genotype, which indicates that a large number of metabolic pathways and cellular processes are involved in LT response. This observation is consistent with earlier reports for Arabidopsis [26,27] and rice [28,29] and raises the question of which sets of genes are directly involved in the development of LT tolerance versus those associated with the general metabolic adjustments that occur with growth at LT.

**Table 2:** Differentially expressed genes

Parameter	*p *< 0.01	*p *< 0.001
Time (T)	26,646	24,670
Genotype (G)	17,472	14,617
GxT interaction (GT)	5,513	3,453
T and G	16,050	12,901
T, G and GT	4,599	2,771

Differentially expressed genes were identified using gene-specific ANOVA with the following model: Y_ijkr _= μ_i _+ G_ij _+ T_ik _+ (GxT)_ijk _+ ε_ijkr _where Y, the response variable, represents the log-2 transformed normalized probeset intensity and μ, represents the grand mean level of expression for each gene. G, T and GxT represents Genotype effect, Time (duration of cold treatment) effect and interaction effect between Genotype and Time, respectively, and ε represents the stochastic error which is assumed to be normally distributed. The indexes *i, j*, *k *and *r *indicate the probeset (gene), genotype, time and biological replications, respectively.

#### 3.2 Genes with GxT interaction and their biological function

Recent studies in wheat indicate that the accumulation of the transcript or protein product of cold-induced genes known to be involved in cold acclimation show strong interaction between genotype and the duration of cold treatment [14,21]. Examination of interactions between factors that influence the results of an experiment can give valuable insights about the behavior of genes that are different from the general effect. Of the 12,901 genes that were differentially expressed with respect to genotype and duration of cold treatment in the present study, 21% (2,771; listed in Additional file 2) showed expression that was strongly influenced by the interaction between these two major factors (GxT interaction). We examined this subset of genes in greater detail to better define their role in the development of LT tolerance and to better understand the mechanisms that underpin LT tolerance gene regulation.

The Gene Ontology (GO) annotations for the biological functions of these genes showed that a majority of them had no known function. Information could be found for only 41% of the genes (1133 out of 2771) identified in these analyses. Genes involved in transport, oxidation-reduction, and stress response were highly represented (Table 3) and genes that respond to stress included several known cold-induced genes.

**Table 3:** Biological functions of the differentially expressed genes (only GO categories with 30 or more genes are shown)

Rank	Biological Function	No. of genes
1	Transport	83
2	Oxidation reduction	75
3	Response to stress	64
4	DNA metabolic process	42
5	Cellular carbohydrate metabolic process	42
6	Small molecule biosynthetic process	42
7	Generation of precursor metabolites/energy	39
8	Cellular amino acid and derivative metabolic process	39
9	Carboxylic acid metabolic process	38
10	Protein amino and phosphorylation	38
11	Regulation of Transcription, DNA-dependent	37
12	Catabolic process	36
13	Cellular nitrogen compound biosynthesis	34
14	Response to chemical stimulus	32
15	Chromatin organization	31
16	Lipid metabolic process	30
17	Cellular Macromolecular complex assembly	30
18	Cellular amine metabolic process	30

#### 3.2.1 Expression profiles of differentially expressed genes with GxT interaction

**Figure 5:**
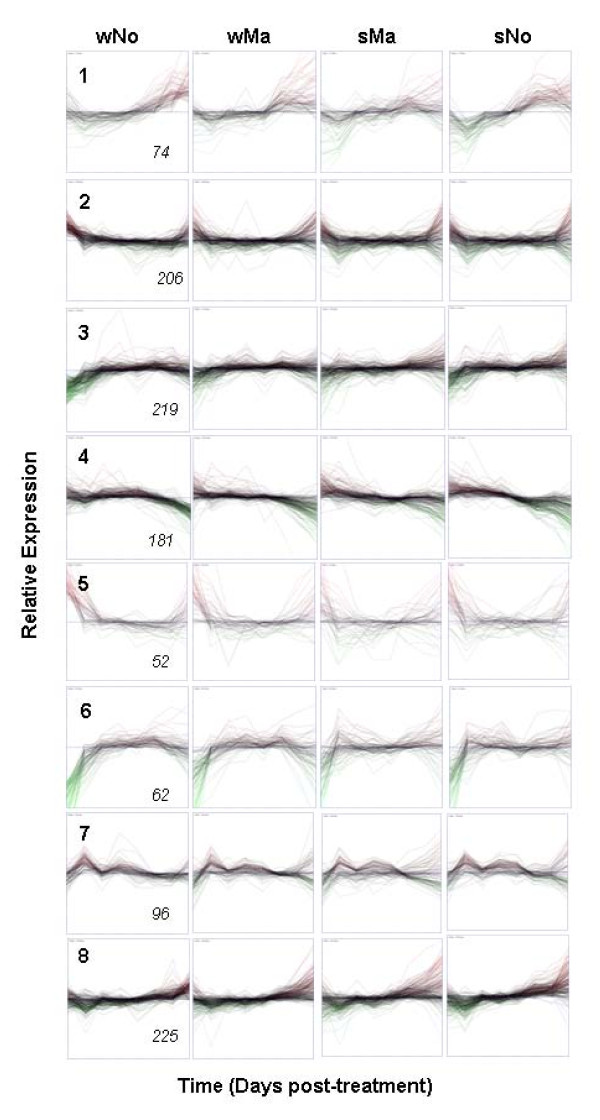
**Clustering of expression profiles of genes that show 2-fold or more change in expression in Norstar and their comparative profiles in other genotypes**. The x-axis in each frame represents duration of cold treatment from 0 to 70 days. The y-axis in each frame represents median centered relative gene expression in log 2. Relative expression was calculated by determining the difference between the maximum and minimum level of expression in the time-series. Cluster ID is indicated by the number on the upper left corner of each frame. The number of genes in each cluster is indicated on the lower right corner of each frame.

Analysis of the expression levels of the 2,771 genes with strong GxT interaction (Table 4) showed that more genes in the spring habit genotypes had a higher frequency of 2-fold or more change in gene expression (61-62%) compared to the winter habit genotypes (41-45%). This suggests that the *vrn-A1 *allele had a dampening effect on LT response. The k-means clustering method was used to identify genes with 2-fold or more change in expression that had similar up- or down-regulation patterns during cold treatment. These genes were grouped into 8 clusters for each genotype. The expression profiles for the 1,137 winter Norstar genes (Figure 5) show that a majority in Cluster 1 are gradually up-regulated and peaked at the later time points. Genes in Clusters 3 and 6 were rapidly up-regulated during the early stage of cold treatment and remained highly expressed. The genes in cluster 7 were also rapidly up-regulated during the initial cold-treatment but showed modulated response thereafter. The genes in Clusters 2, 4, 5, and 8 were down-regulated during the early stage of cold treatment and had different dynamics at later stages. The modulated expression of genes in Cluster 8 was a mirror image of the expression profiles of the genes in Cluster 7.

**Table 4:** Fold-change in level of expression of the 2,771 genes that show GxT interaction at p < 0.001.

Genotype	≥ 1.5×	≥ 2×	≥ 5×	≥ 10×	≥ 25×
winter Norstar	2029	1137	229	90	27
spring Manitou	2382	1723	351	125	20
winter Manitou	2158	1270	237	77	25
spring Norstar	2382	1693	330	100	25

Fold-change in expression was based on the difference between the maximum and minimum levels of gene expression from 0 to 70 days of cold treatment.

When the expression profiles of the same genes from the different genotypes were aligned with respect to winter Norstar (Figure 5) the difference in expression profiles was evident. The expression of the genes in cluster 6 in Norstar was more similar to winter Manitou while this cluster in spring Norstar was more similar to spring Manitou. Cluster 6 contained most of the previously reported cold-induced genes. The expression profiles of the genes in clusters 4 and 8 of spring Norstar were more similar to spring Manitou. Cluster 4 contains several genes involved in photosynthesis. A significant number of unknown genes grouped with genes of known function in each cluster. This transcriptional co-expression with known genes could provide an entry point for the dissection of their biological functions.

#### 3.2.2 Expression of the most dynamically expressed genes

The most dynamically expressed genes in winter Norstar (≥ 25-fold change in expression; Table 4) during the course of the experiment included the vernalization regulatory MADS-box genes *Ta.Vrn-A1 *(Ta.30607.1.A1; [3]) and *Ta.Vrt1 *(Ta.142.1.S1_at; [8,29]) and 10 known cold-responsive genes (COR14a, COR14b, Dehydrin and LEA/RAB-related COR proteins). Several of the known cold-induced genes in winter Norstar were also highly expressed in the other genotypes. In fact the expression of COR14a, a gene specifically up-regulated by LT [30], was induced at a high level in both spring Manitou (203 fold-change) and winter Norstar (177 fold-change) indicating that the regulatory factors needed to up-regulate (see below) the expression of genes correlated with cold acclimation are fully operational in genotypes with spring habit.

#### 3.2.3 Expression of the *Vrn-A1* gene

It was expected that the expression profile of the *Vrn1 *gene on chromosome 5A of the NIL derivatives, winter Manitou and spring Norstar, would be different from their parental lines. Since the *Vrn-A1 *locus from spring Manitou was transferred to spring Norstar, the expression profile of *Vrn-A1 *gene in spring Norstar was predicted to mimic the *Vrn-A1 *gene expression profile from spring Manitou. Conversely, the expression profile of *vrn-A1 *in winter Manitou is predicted to mimic the expression profile of *vrn-A1 *from winter Norstar.

**Figure 6:**
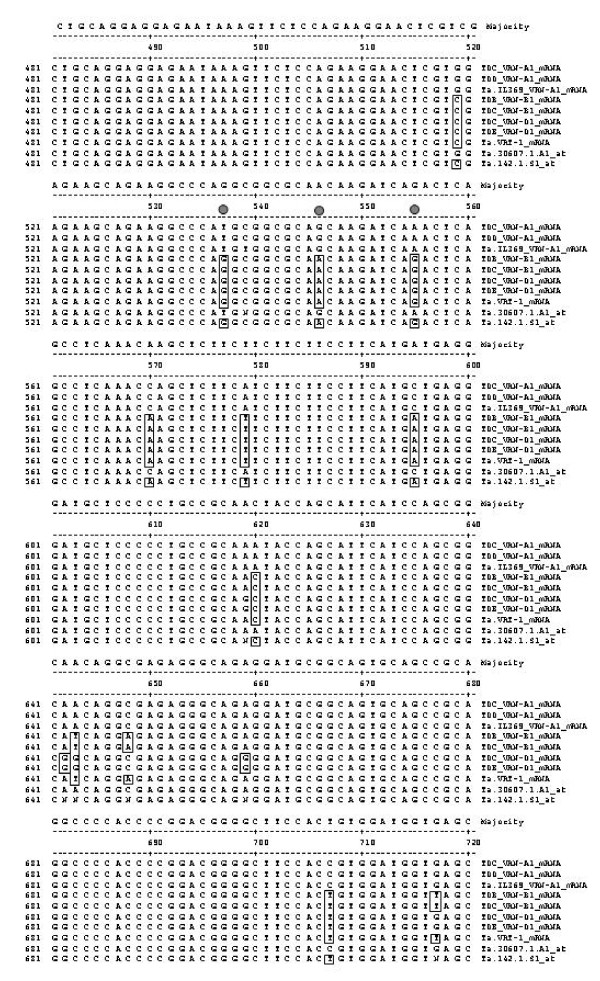
**Comparison of *Vrn-1 *gene sequences from hexaploid wheat *T. aestivum***. Boxed letters indicate divergence from TDC *Vrn-A1 *mRNA sequence. Closed circles below the consensus sequence indicate SNPs covered by the probesets for Ta.30607.1.A1, Ta.142.1.S1_at.

There were three gene specific probesets on the array that represented the *Vrn1 *gene: Ta.30607.1.A1_at, Ta.142.1.S1_at and Ta.Affx.143995.17.A1_at. Closer examination of the DNA sequence of the target genes (Figure 6) from which the probesets were derived indicates that Ta.30607.1.A1_at was most similar to *Ta.Vrn-A1 *(AY747601), a gene sequenced from *T. aestivum *cv. Triple Dirk,[3]; Ta.142.1.S1_at was most similar to *Ta.Vrt-1 *(AY280870) sequenced from *T. aestivum *cv. Norstar [13]; and Ta.Affx.143995.17.A1_at was identical to the *Vrn1 *gene sequenced from *T. monococcum *(EU875079 [30], alignment data not shown). The target sequence used for Ta.Affx.143995.17.A1_at was the anti-sense of EU875079 sequence, thus, the signals derived from the designed probeset might not be valid and it was not analyzed further.

**Figure 7:**
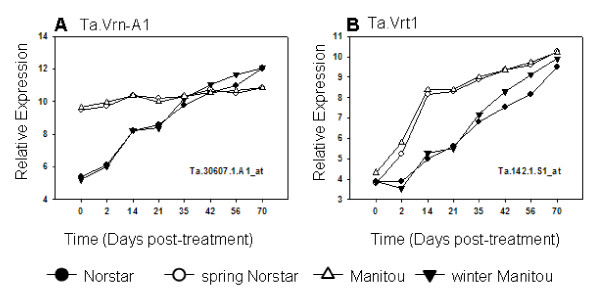
**Expression of vernalization genes *Ta.Vrn-A1 *and *Ta.Vrt1 *in winter Norstar and spring Manitou and their near isogenic lines spring Norstar and winter Manitou acclimated at 6°C for 0 to 70 days**.

As predicted the expression profile of Ta.30607.1.A1_at (*Ta.Vrn-A1*) was swapped between the parental lines and the NIL derivatives (Figure 7). *Vrn-A1 *in spring Norstar is constitutively expressed as in spring Manitou, whereas, *vrn-A1 *in winter Manitou was up-regulated upon cold-treatment and behaved just like *vrn-A1 *in winter Norstar. The high congruence of the expression profiles of *Vrn-A1 *in spring Norstar and spring Manitou and the expression profiles of *vrn-A1 *gene in winter Norstar and winter Manitou indicates that the genetic factors needed to regulate the transcription of *Vrn-A1 *and *vrn-A1 *genes are similar in the two genetic backgrounds.

*Ta.Vrn-A1 *(a.k.a. *WAP1*; [31] and *Ta.Vrt-1 *[13] genes although cloned separately, were hypothesized to encode the same *Vrn1 *gene in the *Vrn-A1 *locus. If this is so, then the expression profile detected by the probesets representing each gene should be similar. As shown in Figure 7, the expression profile of Ta.142.1.S1_at (*Ta.Vrt-1*) was also swapped between the parental lines and the NIL derivatives; however, its expression profile was distinct from the expression profile of Ta.30607.1.A1 *(Ta.Vrn-A1) *indicating that the two genes are different. Closer examination of the alignment of these two sequences with the available sequences for *Vrn-A1*, *Vrn-B1 *and *Vrn-D1 *from hexaploid wheat (Figure 6) showed that Ta.30607.1.A1_at was identical to *Ta.VRN-A1*, whereas, Ta.142.1.S1_at was identical to that of Ta.*Vrn-B1*. There are 15 SNPs that distinguish *Vrn-A1 *from *Vrn-B1 *genes and all the SNPs in *Vrn-A1 *were similar to Ta.30607.1.A1_at and all the SNPs in *Vrn-B1 *were similar to that of the informative SNPs in Ta.142.1.S1_at. There are 6 SNPs that distinguish *Vrn-B1 *from *Vrn-D1*, however, the probeset designed for Ta.142.1.S1_at do not cover them. Thus, the expression profile for Ta.142.1.S1_at does not distinguish the expression between *Vrn-B1 *and *Vrn-D1*.

If *Ta.Vrt1 *encodes Ta.*Vrn-B1*, which is located in chromosome 5B, then its swapped expression profile indicates that it is a downstream target of *Ta.Vrn-A1 *(located in chromosome 5A). The expression profile of *Ta.Vrt1 *in the spring genotypes was up-regulated only after cold treatment suggesting that *Ta.Vrn-A1 *acts together with other cold-induced factors to up-regulate *Ta.Vrt1 *expression. Without the sequence of the *Vrn-A1 *locus, however, we still cannot rule out the possibility that Ta.142.1.S1_at was detecting the expression of another MADS-box gene with sequence similar to *Ta.Vrt1 *located in the *Vrn-A1 *locus.

#### 3.2.4 Expression profiles of known cold-induced genes closely associated with LT tolerance

There are several known cold-induced genes closely associated with LT tolerance that are dynamically expressed and analyses of their expression profiles provided insight into their regulation during cold treatment.

##### Rate of response

**Figure 8:**
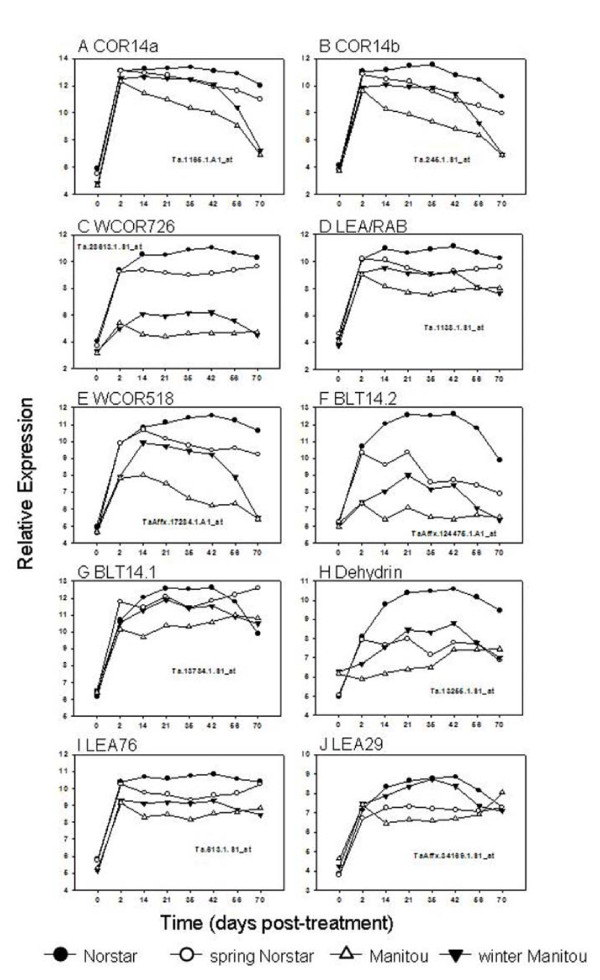
**Expression profiles of known cold-induced genes in winter Norstar and spring Manitou and the near isogenic lines spring Norstar and winter Manitou that showed strong genotype by duration of cold treatment interactions when acclimated at 6°C for 0 to 70 days**.

The initial rate of response (determined by the slope of the change in relative gene expression between 0 and 2 days of cold treatment) of known cold-induced genes was not influenced by the swapped *Vrn1 *locus. As shown in Figure 8, the initial rate of response of these cold-induced genes was similar in winter and spring Norstar and in spring and winter Manitou. This reveals the presence of regulatory genes located outside of the swapped *Vrn1 *locus that control the rate of response of these genes to cold treatment. The initial *rate *of response to cold treatment greatly influences the level of expression of cold-induced genes and genes that exhibit faster rate of up-regulation by acclimation were usually associated with greater cold tolerance (Figure 1). This close relationship among rates of up-regulation of the known cold-induced genes and LT tolerance should not be unexpected as this is likely the reason why they were originally selected for study.

At least two distinct mechanisms regulate the initial *rate *of response of cold-induced genes. The initial rate of response to cold-treatment could be similar (Figure 8A, B, D, G, I and 8J) or different (Figure 8C, E, F and 8H) between Norstar and Manitou genetic backgrounds. When the initial rate of response is different it is usually slower in the Manitou background.

##### Duration of expression

The duration of expression of known cold-induced genes was influenced by the *Vrn1 *locus. The winter habit genotypes (with *vrn-A1 *locus) sustained a high level of gene transcripts for a longer period of time compared to the spring habit genotypes (with *Vrn-A1 *locus). As a result, the expression of the cold-induced genes in winter Manitou was higher than in spring Manitou and lower in spring Norstar compared to winter Norstar. The decline in the level of transcript in spring Manitou and spring Norstar began immediately after 2-days of cold treatment. In contrast, the expression of these genes in winter Norstar and winter Manitou continued to be up-regulated (Figure 8C, D, E, F, H and 8J) or sustained at high level (Figure 8A, B and 8I) until at the point of vernalization saturation. This observation supports the developmental model proposition that the *duration *of expression of LT tolerant genes is controlled by genes that determine the length of the shoot apical meristem vegetative stage.

#### 3.2.5 Expression of other dynamically cold-responsive genes

The expression of jacalin-like lectin (Ta.7388.2.S1_a_at) was initially down-regulated in all the genotypes but was up-regulated in the spring habit lines after two days of cold treatment. The gene continued to be down-regulated in the winter-habit lines until vernalization saturation. A jacalin-like lectin domain containing protein (a.k.a.VER2) previously isolated in winter wheat [32] has been shown to be involved in vernalization. Antisense inhibition of VER2 delayed heading in transgenic hexaploid wheat. Thus, the prolonged down-regulation of jacalin-like lectin could be contributing in the delayed acquisition of competence to flower in the winter-habit lines.

**Figure 9:**
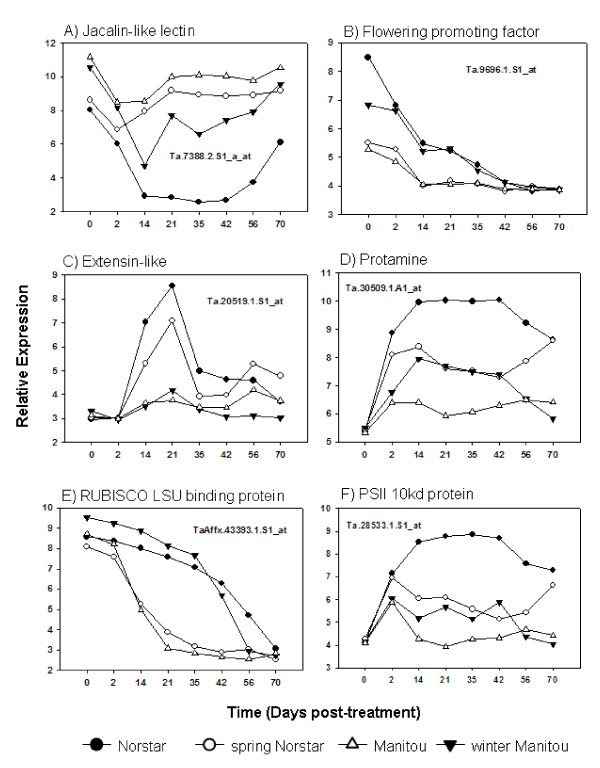
**Expression profiles of selected most dynamically expressed genes in winter Norstar and spring Manitou and the near isogenic lines spring Norstar and winter Manitou acclimated at 6°C for 0 to 70 days**.

The expression of a flower promoting factor (FPF; Ta.9696.1.S1_at) was down-regulated by cold treatment (Figure 9B) in all four genotypes. The level of expression of this gene was also swapped between the winter and spring-habit genotypes. The overexpression of the FPF under inductive condition led to early flowering in tobacco [33]. However, under non-inductive conditions the FPF overexpressing lines bolted but did not flower. It is also possible that an inductive condition to flower is required for the up-regulation of this gene in wheat.

The expression profile of an extensin-like protein (Ta.20519.1.S1; Figure 9C) was similar in winter Norstar and spring Norstar. The gene was induced after 2 days of acclimation and reached its maximum expression by 21 days of cold treatment. Extensin has been shown to be induced by cold treatment in pea seedlings [34] and speculated to be involved in the strengthening of the cell wall to prevent the cavitation of the cell during cold induced dehydration.

This is the first time that protamine (Ta.30509.1.A1_at; Figure 9D) has been shown to be induced by cold in wheat. Its role in the development of cold tolerance is unclear, however, in animal studies protamine has been shown to be involved in the maintenance of high order chromatin structure in trout and sea urchin sperm nuclei. It accumulates late in spermatogenesis and displaces or augments the germ cell histones to stabilize the highly condensed chromatin. It is hypothesized to play a role in global chromatin reorganization and accessibility of histone variant regions [35]. Protamine could also be involved in the epigenetic regulation of the *Vrn-A1 *locus as it has also been postulated that maintenance of an active chromatin state at *Vrn1 *is the basis for epigenetic memory of vernalization in cereals [36].

#### 3.3 Differentially expressed genes in genotypes with neutralized *Vrn-A1* locus

Comparison of gene expression in genotypes where the *Vrn-A1 *is similar or neutralized is expected to identify genes regulated by factors outside of the swapped *Vrn-A1 *locus. There were 7,808 genes (at *p *< 0.001) differentially expressed between winter Norstar and winter Manitou with respect to genotype and duration of cold treatment. The expression of 25% of these genes (1,952) showed strong interaction between these two factors. Twenty-three of these genes were dynamically expressed (> 25 fold-change) in winter Norstar, the identities of which were similar to those shown in Table 5 except for 4 missing genes. The expression profiles of these 4 genes in spring Norstar and spring Manitou were also very similar: *Ta.Vrn-A1 *(Ta.30607.1.A1_at; Figure 7A), RUBISCO LSU binding protein (TaAffx.43393.1.S1_at; Figure 9E), LEA29 protein (TaAffx.34169.1.S1_at; Figure 9J)) and flower promoting factor (Ta.9696.1.S1_at; Figure 9B) indicating that the genes regulating their expression were swapped between the winter and spring habit genotypes. Since the *Vrn-A1 *locus is the same in winter Norstar and winter Manitou, the swapped expression profiles of RUBISCO LSU binding protein, LEA29 and flower promoting factor indicates that these genes are either located in the *Vrn-A1 *locus or they are controlled by outside factors that are regulated by the *Vrn-A1 *locus. If they are located outside of the *Vrn-A1 *locus then these genes are strong candidates for direct involvement in the development of LT tolerance.

**Table 5:** Winter Norstar genes that exhibit 25-fold or more change in expression during cold-treatment and show significant interaction between genotype and duration of cold treatment.

Probeset ID	Description	No	Ma	wMa	sNo
Ta.1165.1.A1_at	cold-responsive protein COR14a	177	203	231	189
Ta.245.1.S1_at	cold-responsive protein COR14b	173	61	67	129
Ta.28613.1.S1_at	cold acclimation protein WCOR726	124	5	7	61
Ta.1138.1.S1_at	cold-responsive LEA/RAB-related COR protein	117	31	53	47
Ta.30607.1.A1_at	MADS-box protein Ta.VRN-A1	98	2	117	3
TaAffx.17284.1.A1_at	Cold acclimation protein WCOR518	93	10	34	66
TaAffx.124475.1.A1_at	low-temperature responsive gene BLT14.2	89	3	7	17
Ta.13784.1.S1_at	low-temperature responsive gene BLT14.1	89	24	42	71
Ta.23327.1.S1_at	Unknown	61	5	10	30
Ta.13255.1.S1_at	Dehydrin	50	3	6	8
Ta.142.1.S1_at	MADS-box protein TaVRT-1	49	59	83	90
Ta.20519.1.S1_at	Extensin-like	48	2	2	17
TaAffx.43393.1.S1_at	Rubisco LSU	46	72	110	48
Ta.29534.1.S1_at	senescence-specific cysteine protease SAG12	45	232	238	197
Ta.7388.2.S1_a_at	Jacalin-like lectin domain containing protein	44	6	57	5
Ta.13239.1.S1_at	glycine-rich cell wall protein homolog	41	28	29	28
Ta.19303.1.S1_at	Unknown	40	11	11	38
Ta.30798.3.S1_at	gamma-VPE	36	21	25	25
Ta.613.1.S1_at	LEA76 homologue type2	32	15	18	22
TaAffx.34169.1.S1_at	LEA29 D-29 protein	32	11	22	11
Ta.865.2.A1_at	arginine/serine-rich protein, putative	32	19	24	25
Ta.9000.1.S1_at	beta-fructosidase	29	38	36	29
Ta.27389.1.S1_at	Gamma-1-purothionin	28	19	40	35
Ta.28533.1.S1_at	Photosystem II 10 kDa polypeptide, chloroplast	27	4	4	6
Ta.18720.1.S1_a_at	Gamma-thionin	26	42	88	65
Ta.30509.1.A1_at	Protamine	26	2	5	9
Ta.9696.1.S1_at	Flowering promoting factor-like 1	25	3	8	3

Fold-change in expression was based on the difference between the maximum and minimum levels of gene expression from 0 to 70 days of cold treatment.

There were 5,982 genes (at *p *< 0.001) that were differentially expressed between spring Norstar and spring Manitou with respect to genotype and duration of cold treatment. The expression of 29% of these genes (1,717) showed strong interaction between these two factors. In both these spring genotypes, the cold-responsive gene COR14a was the most dynamically expressed gene.

**Figure 10:**
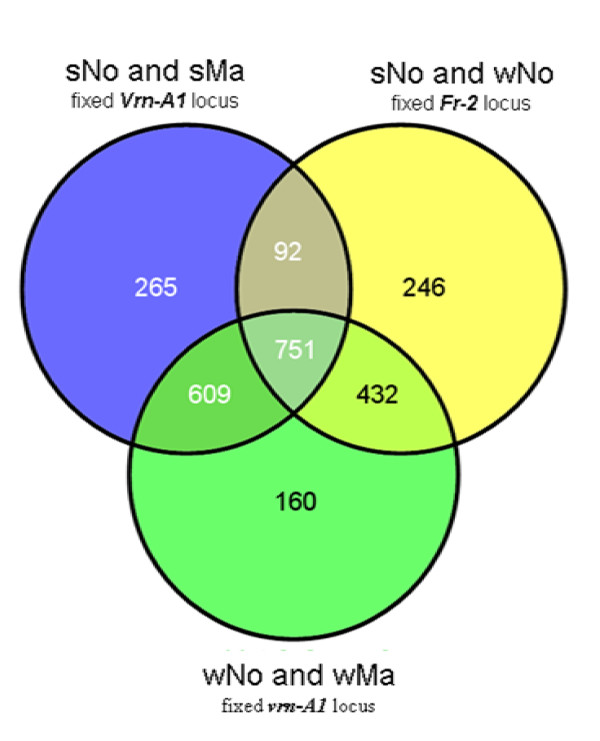
**Genes controlled by the *Vrn-A1 *and *Fr-2 *loci**. Venn diagrams showing the statistically significant (*P *< 0.001) differentially expressed genes that show GxT interaction in genotypes with neutralized *Vrn-A1 *or *Fr-2 *locus.

As shown in Figure 10, there were 265 genes that were differentially expressed specifically only when the *Vrn-A1 *(spring) locus was neutralized. A significant number of these genes are involved in chromatin remodeling e.g. Curly leaf like gene, chromatin remodeling 1 and 17, several core histone genes, methyltransferase1 and histone acetyltransferase genes and several DNA binding proteins like HTB2, HTB11, HTA2, 6 and 12. There were 160 genes that were specifically associated with a neutralized *vrn-A1 *(winter) locus and a significant number of these genes are transcription factors and genes involved in signal transduction, transport and oxidation-reduction pathways.

#### 3.4 Differentially expressed genes in genotypes with neutralized Fr-2 locus

Genes that were differentially expressed in genotypes with neutralized *Fr-2 *locus in the Manitou and Norstar backgrounds were expected to be directly or indirectly influenced by the *Vrn-A1 *locus. They were also predicted to be part of pathways where the vegetative/reproductive transition acts as a key switch in LT gene expression. There were 4,411 genes (at *p *< 0.001) that were differentially expressed in winter and spring Norstar grown under the cold treatment time series used in this study. The expression of 34% of these genes (1,521) showed GxT interaction and 246 of these genes were strongly influenced by the *Vrn-A1 *locus (Figure 10). Of this group, 26 genes were dynamically expressed (> 25 fold-change) in winter Norstar. These genes were identical to those listed in Table 5 except that Ta.20519.1.S1_at (extensin-like gene) was not present. As shown in Figure 9C, Ta.20519.1.S1_at expression profile was similar in winter Norstar and spring Norstar and not swapped in the spring and winter habit genotypes.

There were 4,064 genes (at *p *< 0.01) that were differentially expressed in winter and spring Manitou, 53% (2,164) of which showed GxT interactions. However, there were no differentially expressed genes between spring and winter Manitou that showed GxT interaction at a higher level of confidence (*p *< 0.001). These observations suggest that the *Vrn-A1 *locus had a much variable influence on gene expression on the cold hardy Norstar than the more cold sensitive Manitou genetic background.

#### 3.5 Mapping of differentially expressed genes in Rice and Brachypodium genome

The swapped *Vrn-A1 *locus in Norstar and winter Manitou was determined to be less than 37 cM using 678 SSR markers mapped on a 152 double haploid Norstar × winter Manitou mapping population [4]. There are a number of possible reasons for the low mapping resolution of the swapped *Vrn-A1 *locus, one of which could be the limited number of markers available at the time to these studies. It is also possible that the swapped region was already highly similar (with very low polymorphism) in the two parental genotypes. To assist in determining if the swapped expression profiles were due to genes closely linked to *Vrn-A1 *or genes located elsewhere that were being regulated by *Vrn-A1 *we determined the location of the 2771 differentially expressed genes in the syntenic region of Brachypodium and rice genomes. We used the dataset provided in HarvEST: WheatChip (http://harvest.ucr.edu/ which contains for each of the Affymetrix probeset target sequence the best BLASTX hits from the rice genome ( MSU version 6; January 2009) and Brachypodium ( Phytozome Bradi 1; May 2009) gene models and their genome location allowing us to filter for the differentially expressed genes located in the syntenic region in wheat.

Of the 2771 differentially expressed genes that showed GxT interaction, there were 2352 (84.9%) and 2283 (82.45%) that can be mapped to the Brachypodium and rice genomes, respectively (see Additional file 2). The *Vrn1 *gene homologue is located in chromosome 1 in Brachypodium and in chromosome 3 in rice [37,38]. There were 708 differentially expressed genes that mapped to Brachypodium chromosome 1 and 284 of these also mapped to rice chromosome 3. Brachypodium chromosome 1 was assembled from scaffold 0 (38 Mb) and scaffold 1 (37 Mb), which formed the top and bottom part of the chromosome. There were only 145 differentially expressed genes that can be mapped to scaffold 0 of Brachypodium where the *Vrn1 *homologue is located. Analysis of the mapping data suggests that these genes are located in 18 Mb and 34 Mb of the syntenic regions in Brachypodium and rice genomes, respectively. Among the dynamically expressed genes in Norstar, aside from those encoding the *Vrn1 *genes only Ta.9696.1.S1_at (flowering promoting factor-like 1) mapped to this region. Our data indicate that the majority of the differentially expressed genes are not linked to the *Vrn-A1 *locus and those that exhibit swapped expression between winter and spring habit genotypes are potential direct or indirect targets of the *Vrn-A1 *locus.

## 4. Conclusion

### 4.1 Phenology of wheat response to cold

The progress to the vegetative/reproductive transition (VRT) during vernalization can be revealed by growth in an inductive (warm) environment. Our data indicate that the SAM competence to flower and the morphological transition to a reproductive meristem can be uncoupled. Development of SAM of the four genotypes was delayed (remained morphologically in stage 0 - vegetative) by low temperature even when competent to flower once they were transferred to an inductive environment.

Cold-acclimation in cereals is cumulative and peaks at the point of VRT. The SAM increasingly becomes sensitive to low temperature after VRT even before cold sensitive reproductive structures are formed. This indicates that spring habit genotypes are likely to be cold sensitive not because of the early formation of cold susceptible reproductive structures during winter but due to an altered molecular/physiological state of the SAM after VRT is reached. Our data shows that after VRT the plant continuous to lose the ability to cold acclimate such that at 70 days after cold treatment the winter habit genotypes were almost as cold sensitive as the untreated spring habit genotypes. These observations indicate that identification of the factors regulating VRT is critical to understanding and controlling low temperature adaptation in wheat. The molecular data presented in this report showed that the different state of the SAM before and after VRT can be distinguished based on the differences in their gene expression - allowing us to have a window by which to enter and dissect this phase transition.

### 4.2 Genetic control of LT response in wheat

The large number of genes that changed in expression during acclimation/vernalization of the four genotypes considered in this study once again emphasizes the complexity of the genotype × environment × stage of phenological development interactions and the metabolic pathways and cellular processes that determine LT adaptation in wheat. The identification of genes with expression that are influenced by the interaction of the genotype and the duration of treatment allowed us to enrich for genes that may be directly involved in the development of LT tolerance.

Analyses of the expression profiles of the known cold-induced genes provided novel insights into the mechanisms that underpin their regulation. We confirmed the constitutive expression of the *Ta.Vrn-A1 *gene in the spring genotypes and demonstrated that it is distinct from *Ta.Vrt1*. Our data indicates at least two regulatory networks control the level of expression of many of the known cold-responsive genes. Consistent with the phenotypic response of the shoot apical meristem to cold treatment, the molecular evidence shows that the initial rate of response of these genes was not controlled by the *Vrn-A1 *locus. However, the duration of the expression of these genes was regulated by the *Vrn-A1 *locus and the VRT is a key factor affecting LT tolerance gene expression. These observations provide strong molecular support for the developmental model of LT tolerance gene regulation in wheat [1]. While more research is needed to determine how the sets of genes we have reported affect the wheat plants response to cold, our findings provide directions for future attempts to expand our understanding of low-temperature adaptation in cereals.

## Authors' contributions

BF, RC, SG and DLC designed the experiment. BF carried out the phenotypic analysis (LT_50 _and FLN determination) and cold-treatment of the biological materials. SG isolated the total RNA. DLC and FY analyzed the microarray data. DLC and BF interpreted the results. RC, OA and DLC coordinated the study. DLC wrote the manuscript. All authors read and approved the final manuscript.

## Supplementary Material

Additional file 1 **Correlation of variance heatmap and dendogram**. Correlation of variance heatmap and dendogram for the 96 × 96 matrix of pairwise comparisons between samples. Click here for file

Additional file 2 **Differentially expressed genes**. Annotated list of 2771 differentially expressed genes that show GxT interaction. Click here for file
