# Supplementary figures and images for: Genome-wide gene expression analysis supports a developmental model of low temperature tolerance gene regulation in wheat (Triticum aestivum L.)
Source: BMC Genomics. 2011 Jun 7;12:299. doi: 10.1186/1471-2164-12-299 (PMC3141665; doi:10.1186/1471-2164-12-299)

A 96X96 comparison of sample coefficient of variation correlation heatmap

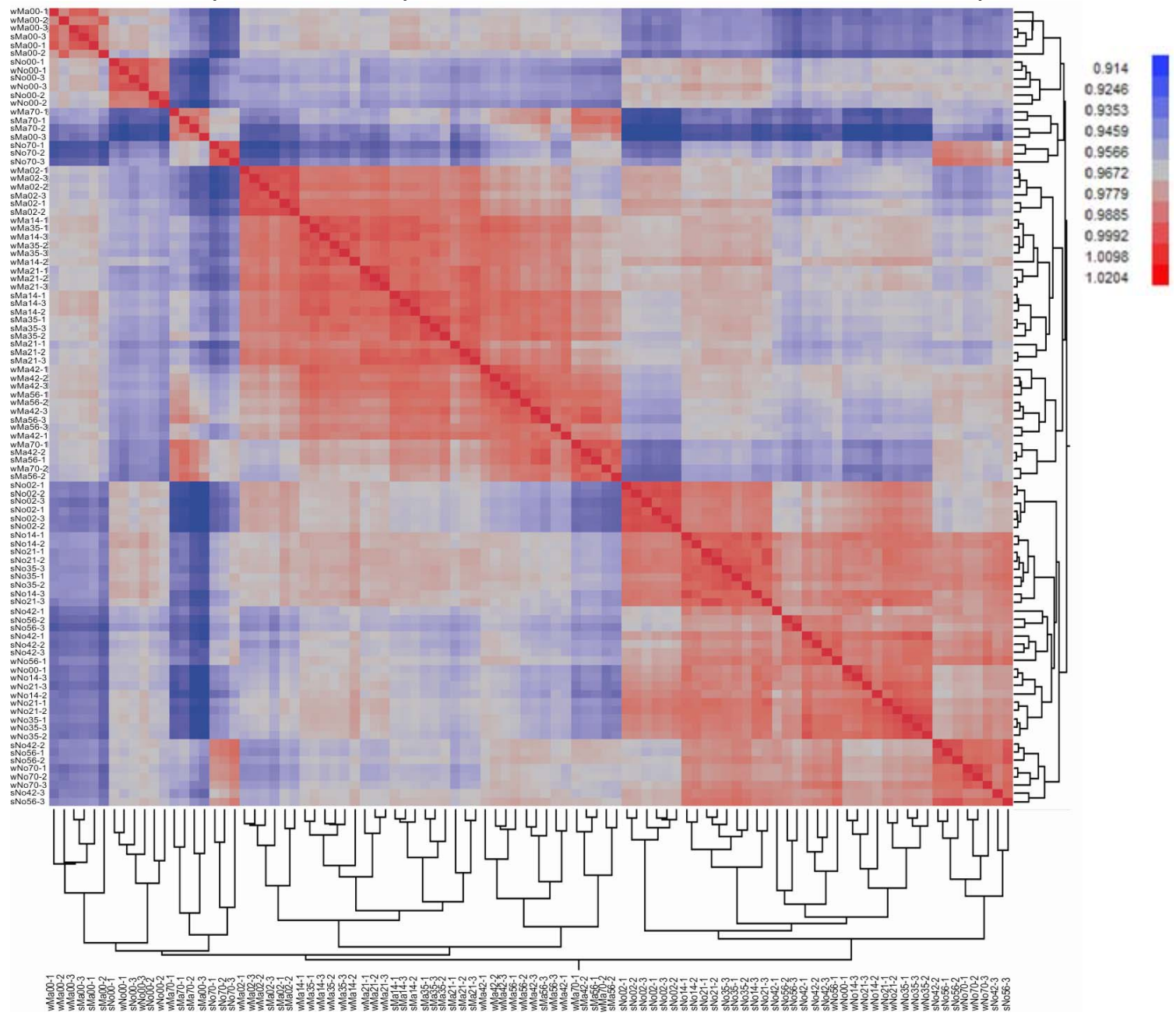

Supplement: Additional file 1 — Correlation of variance heatmap and dendogram. Correlation of variance heatmap and dendogram for the 96 × 96 matrix of pairwise comparisons between samples. [file 1471-2164-12-299-S1.PDF]
